# Longitudinal Cognitive Evolution in Alcohol Use Disorder Patients: Role of Inflammation, Time of Abstinence and Apolipoprotein Profile

**DOI:** 10.1111/adb.70174

**Published:** 2026-07-01

**Authors:** Berta Escudero, Ricardo Olmos, Francisco Arias, Laura Orio

**Affiliations:** ^1^ Department of Psychobiology and Behavioral Science Methods, Faculty of Psychology Complutense University of Madrid Pozuelo de Alarcón Spain; ^2^ Instituto de Investigación Sanitaria Hospital Universitario 12 de Octubre (imas12) Madrid Spain; ^3^ Research Network in Primary Care in Addictions (RIAPAd) Madrid Spain; ^4^ Department of Social Psychology and Methodology, Faculty of Psychology Autónoma University of Madrid C/Iván Pavlov Spain

**Keywords:** alcohol, apolipoproteins, cognition, inflammation, LPS

## Abstract

Alcohol use disorder (AUD) is linked to cognitive deficits that can persist even after prolonged abstinence. Research highlighted associations between plasma apolipoproteins and cognition in AUD. This study examines the longitudinal evolution of plasma apolipoproteins and inflammation in AUD patients during early and prolonged abstinence and their association with cognitive recovery. Thirty‐three AUD patients from an outpatient hospital alcoholism programme were evaluated at baseline (*t* = 0), 6 months (*t* = 1) and 12 months (*t* = 2), along with 34 healthy controls. Cognitive performance was assessed using the TEDCA test, which measures general cognitive function (GCF). Biological assessments included plasma pro‐inflammatory biomarkers (LPS and LBP) and several apolipoproteins (APOAI, APOAII, APOB, APOCII, APOE, APOJ and APOM). AUD patients showed elevated plasma LPS, APOAI, APOE, APOJ and downregulated APOM, which all normalized at *t* = 1, whereas cognitive improvement was significant at t = 2 compared to controls. Mixed‐effects models including all covariates and within‐between person decomposition, followed by correlation matrix and reduced mixed models sensitivity analysis support robust associations between better GCF and (1) the duration of abstinence and (2) within‐person reductions in LPS, although apolipoprotein changes should be considered exploratory at this level. Prolonged abstinence in AUD patients normalizes plasma peripheral inflammation and apolipoprotein levels and improves cognition. Although APOAI and APOM showed opposite trajectories, as opposite biomarkers in AUD diagnosis reported previously, only the duration of abstinence and normalization of within‐person blood LPS levels over time emerged as predictors of cognitive recovery.

## Graphical abstract

Thirty‐three AUD patients enrolled in an Alcohol Detoxification Program at Hospital Universitario 12 de Octubre (Madrid, Spain) and 34 healthy controls were followed for 12 months. AUD patients exhibited impaired general cognitive functioning, elevated plasma LPS and altered apolipoproteins (APOAI, APOAE, APOAJ, APOAM), which progressively normalized during abstinence (6 months), whereas cognitive improvement emerged later (12 months). Mixed‐effects models and correlation matrix showed that longer abstinence and reductions in plasma LPS were predictors of better general cognitive functioning.

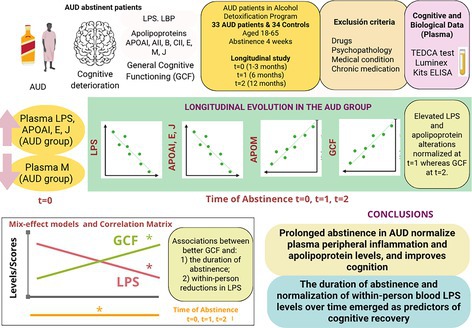

## 1 Introduction

Alcohol use disorder (AUD) remains a major global health concern, not only because of its high prevalence and morbidity but also because of its strong association with persistent cognitive impairments that significantly compromise functional outcomes and treatment adherence. Cognitive deficits are observed in 30%–80% of patients undergoing treatment, encompassing domains such as memory, executive function (EF) and visuospatial processing [1, 2, 3, 4]. These impairments may persist despite sustained abstinence and are frequently underdiagnosed due to reliance on time‐intensive neuropsychological evaluations with limited accessibility.

Emerging research underscores the contribution of peripheral inflammation and the gut–brain axis in the cognitive dysfunction associated with AUD. Chronic alcohol consumption disrupts intestinal permeability, facilitating the translocation of bacterial components—most notably lipopolysaccharide (LPS)—into systemic circulation, a condition termed ‘leaky gut’ [5]. Elevated plasma LPS levels in AUD activate the innate immune response, propagating peripheral inflammation and neuroinflammation, which in turn have been linked to cognitive decline [4, 6, 7, 8]. Plasma LPS may bind to lipopolysaccharide binding protein (LBP), an acute‐phase protein that facilitates the transfer of LPS to immune cell surface receptors (CD14/TLR4 complex) [9], and it also binds to apolipoproteins, which may modulate its function [10, 11].

Apolipoproteins, key structural and functional components of lipoproteins, are gaining attention as potential modulators of the alcohol‐induced inflammatory cascade [4, 11]. Beyond their classical roles in lipid transport, certain apolipoproteins, such as APOAI and APOE, may interact with LPS, potentially influencing its systemic clearance or delivery to the brain [10, 12, 13, 14, 15]. Preclinical studies have demonstrated that LPS can form aggregates with APOAI and APOB within the rat brain following binge alcohol exposure, with region‐ and sex‐specific differences in inflammatory signalling [11]. In clinical populations, we showed upregulations of multiple plasma apolipoproteins, including APOAI, APOB, APOE and APOJ, and downregulation of APOAM in AUD patients that underwent an alcohol dishabituation programme during early abstinence (1–3 months), with no significant sex differences [4]. Interestingly, we identified divergent roles of APOAI and APOM in these abstinent AUD patients, where elevated APOAI and reduced APOM plasma concentrations were, respectively, associated with greater inflammatory burden and poorer cognitive performance [4]. We also identified APOAI as a common apolipoprotein upregulated in rat and mice models of alcohol abuse and in AUD patients during early abstinence, finding correlations between the plasma levels and memory performance in animals and humans [16].

Despite these cross‐sectional findings, longitudinal data elucidating how different peripheral apolipoprotein levels evolve over the course of abstinence during the dishabituation programme and their relationship to cognitive trajectories in AUD are lacking. For example, we have previously found increases in plasma reelin in AUD patients, a biomarker that correlates with poor cognition especially in APOE4 carriers that evolve during abstinence [2]. Thus, longitudinal studies are critical to establish whether these peripheral biomarkers reflect state‐dependent inflammatory processes and/or represent predictors of long‐term cognitive outcomes.

The present study seeks to address this gap by examining the longitudinal profiles of several apolipoproteins and their association with cognitive changes in the same cohort of abstinent AUD patients evaluated at baseline (*t* = 0), 6 months (*t* = 1) and 12 months (*t* = 2). We hypothesize that patients with AUD undergoing sustained abstinence and treatment will exhibit significant longitudinal changes in inflammatory biomarkers (LPS and LBP) and plasma apolipoproteins (APOAI, APOAII, APOB, APOCII, APOE, APOJ and APOM). Specifically, we propose that cognitive improvements over time will correlate with reductions in peripheral inflammation and normalization of apolipoprotein profiles. Given the exploratory nature of the study, the biomarker analyses are examined as candidate correlates of cognitive recovery rather than as validated predictors.

## 2 Methods

### 2.1 Participants and Study Design

**FIGURE 1:**
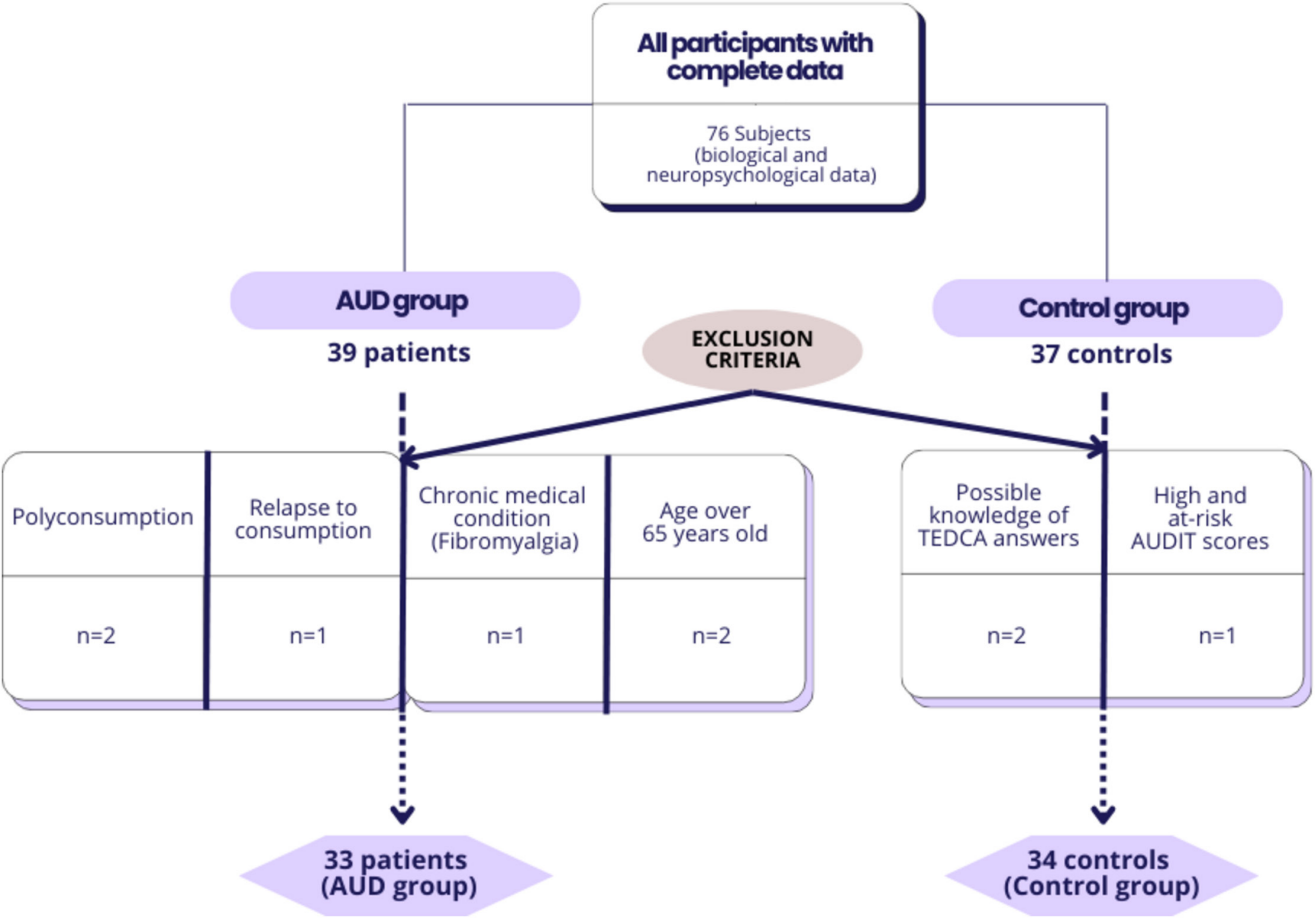
Flowchart of participants recruited for the study.

The initial sample included 76 Caucasian adults (Figure 1). The AUD group (*n* = 39) was recruited from an outpatient treatment programme including pharmacological and psychological intervention for alcohol dependence at *Hospital Universitario 12 Octubre* (Madrid, Spain). The control group (*n* = 37) included healthy controls from the general population (random sampling; Supporting Information Section 1.1). All participants provided written informed consent prior to inclusion.

The study was designed as a prospective longitudinal study, with baseline time (*t* = 0, recruitment at 1–3 months of abstinence), *t* = 1 (6‐month follow‐up) and *t* = 2 (12‐month follow‐up). The experimental subject's loss was four patients and two controls between *t* = 0 and *t* = 1 and one patient between *t* = 1 and *t* = 2.

### 2.2 Inclusion/Exclusion Criteria

#### 2.2.1 Inclusion Criteria

Age: 18–65 years old. AUD diagnosis by DSM‐5 and at least 4 weeks of abstinence before baseline assessment (dishabituation phase).

#### 2.2.2 Exclusion Criteria

History of abuse/dependence to other drugs (including alcohol in control group), except tobacco; psychiatric comorbidity or concomitant diagnosed psychological disorder (emotional symptomatology—mild anxiety/depressive symptoms—was allowed); chronic medical condition; infectious diseases (for instance, HIV infection or hepatitis); liver disease (chronic hepatitis, cirrhosis or liver cancer); and chronic use of anti‐inflammatory medication.

See clinical and psychological assessment methodology used to ensure compliance with inclusion/exclusion criteria in Supporting Information Section 1.2.

After corroborating inclusion/exclusion criteria, the final sample comprised 33 AUD patients (24 men and 9 women) and 34 controls (16 men and 18 women) at *t* = 0 (Table 1).

**TABLE 1:** Characteristics of the AUD and control groups at *t* = 0, *t* = 1 and *t* = 2.

Variable		*t* = 0			*t* = 1			*t* = 2		
		AUD (*N* = 33)	Control (*N* = 34)	*p*	AUD (*N* = 29)	Control (*N* = 32)	*p*	AUD (*N* = 28)	Control (*N* = 32)	*p*
Age [mean (SD)]	Years	49.36 (7.41)	37.41 (12.61)	**< 0.01** ^a^	50.03 (7.97)	36.66 (11.98)	**< 0.01** ^a^	50.39 (6.91)	36.66 (11.98)	**< 0.01** ^a^
BMI [mean (SD)]	kg/m^2^	26.33 (4.84)	24.44 (3.90)	> 0.05^b^	27.16 (4.53)	24.89 (4.50)	> 0.05^a^	26.73 (4.87)	24.89 (4.50)	> 0.05^a^
Sex [*N* (%)]	Women	9 (27.3)	18 (52.9)	0.05^b^	8 (27.6)	16 (50.0)	0.05^b^	8 (28.6)	16 (50.0)	0.05^b^
	Men	24 (72.7)	16 (47.1)		21 (72.4)	16 (50.0)		20 (71.4)	16 (50.0)	
Education [*N* (%)]	Basic	12 (36.4)	5 (14.7)	> 0.05^b^	9 (31.0)	4 (12.5)	> 0.05^b^	9 (32.1)	4 (12.5)	> 0.05^b^
	Higher	21 (63.6)	29 (85.3)		20 (69.0)	28 (87.5)		19 (67.9)	28 (87.5)	
Current work status [*N* (%)]	Employed	26 (78.8)	32 (94.1)	> 0.05^b^	24 (82.8)	30 (93.8)	> 0.05^b^	23 (82.1)	30 (93.8)	> 0.05^b^
	Unemployed	7 (21.2)	2 (5.9)		5 (17.2)	2 (6.3)		5 (17.9)	2 (6.3)	
Current smoking status [*N* (%)]	Yes	20 (60.6)	16 (47.1)	> 0.05^b^	16 (55.2)	13 (40.6)	> 0.05^b^	14 (50.0)	13 (40.6)	> 0.05^b^
	Former	5 (15.2)	4 (11.8)		1 (3.4)	4 (12.5)		2 (7.1)	4 (12.5)	
	No	8 (24.2)	14 (41.2)		12 (41.4)	15 (46.9)		12 (42.9)	15 (46.9)	

*Note:* Anthropometric measures: weight, height and body mass index (weight in kg/height squared). Education (basic means no education; higher means education in college degree). The significant values (*p* < 0.05) are denoted by bold entries in the table.

Abbreviations: AUD = alcohol use disorder, BMI = body mass index, *N*= total of cases, SD = standard deviation, *t* = 0 (recruitment 1–3 months of abstinence), *t* = 1 (6‐month follow‐up), *t* = 2 (12‐month follow‐up).

^a^ *p* value from Student's *t*‐test.

^b^ *p* value from Fisher's exact test.

### 2.3 Alcohol Abuse Variables and Liver Status at Baseline

A semi‐structured interview was administered to both groups, providing insights into their alcohol use history and the use/abuse of other substances (Supporting Information Section 1.2 and Table S1).

In the AUD group, liver status was evaluated using standard clinical practices, analysing levels of alanine aminotransferase (ALT), aspartate aminotransferase (AST), gamma‐glutamyl transferase (GGT), alkaline phosphatase (ALP) and bilirubin. When liver damage was suspected, patients underwent liver ultrasound examinations in the Digestive Department. No liver analyses were conducted in the control group, assuming a sample of healthy controls.

### 2.4 Neuropsychological and Clinical Evaluation

Cognitive function was assessed using the ‘Test of Detection of Cognitive Impairment in Alcoholism’ (TEDCA), a screening test specifically validated for AUD [17]. TEDCA provides a Global Cognitive Functioning (GCF) score and evaluates three core domains: Visuospatial Cognition, Memory/Learning and EF (see Supporting Information Section 1.3 and Table S2).

The clinical evaluation included comprehensive data on sociodemographic and pharmacological variables. The AUD and control groups were assessed for depressive and anxiety symptomatology (in the absence of a psychiatric diagnosis) by using the Beck Depression Inventory‐II (BDI‐II) and the Beck Anxiety Inventory (BAI), respectively (Supporting Information Section 1.3).

Neuropsychological and clinical assessments were repeated at *t* = 1 and *t* = 2 for the AUD group and at *t* = 1 for the control group to account for potential learning effects resulting from repeated test administration.

### 2.5 Blood Sample Processing

Venous blood samples were collected between 8:00 and 10:00 a.m., following a fasting period of 8–12 h. Blood was drawn by venipuncture into BD vacutainer tubes that contained K2‐EDTA anticoagulant (BD, Franklin Lakes, NJ, USA) and processed immediately to obtain plasma following our previous study [2] (Supporting Information Section 1.4). All samples were registered (code number) and stored at −80°C until further immunoassay analysis.

Sample collection was repeated at *t* = 1 and *t* = 2 in the AUD group. In the control group, blood was collected only at baseline (*t* = 0) because biomarker levels in the control group were used as reference ranges for inflammation and apolipoprotein levels in no disease conditions.

### 2.6 Biomarker Quantification

Plasma levels of LPS, LBP, APOJ and APOM were measured using commercially available enzyme‐linked immunosorbent assay (ELISA) kits. Levels of apolipoproteins APOAI, APOAII, APOB, APOCII and APOE were quantified using the MAGPIX multiplex immunoassay technology (xMap; Luminex Corporation), following the manufacturer's instructions (Supporting Information Section 1.5).

### 2.7 Statistical Analysis

Comparisons between the AUD group and control group were performed using Fisher's exact test, chi‐square test or Student's *t*‐test, depending on the statistical assumptions for each test. The results are presented as the number and percentage of subjects [*N* (%)] or as the mean and standard deviation [mean (SD)].

To analyse longitudinal changes and predictive relationships across timepoints, a series of linear mixed‐effects models were applied (see Supporting Information Section 1.6): Model 1 examined changes in GCF over time (*t* = 0, *t* = 1 and *t* = 2) within the AUD group. Model 2 evaluated longitudinal changes in plasma biomarkers (LPS, LBP and apolipoproteins) across the abstinence period. Model 3 assessed whether changes in biomarkers that showed significant variation in Model 2 (LPS, APOAI, APOB, APOE, APOJ and APOM) were associated with cognitive changes (GCF) over the same abstinence process.

The multivariate biomarker model was complemented with the following: (a) Correlation matrix analyses to assess potential collinearity among biomarkers. To distinguish intraindividual biomarker changes from stable between‐participant differences, time‐varying biomarkers included in the mixed‐effects models were decomposed into within‐person and between‐person components. Within‐person components were computed by person‐mean centring each biomarker across repeated assessments, whereas between‐person components corresponded to each participant's average biomarker level. Both components were included in the multivariable model predicting GCF. (b) Sensitivity analyses using reduced mixed‐effects models. All models included sex, age, education, time of abstinence, LPS (within) and LPS (mean) and each apolipoprotein and its person‐level mean were added separately. (c) Interval‐specific reduced mixed‐effects models comparing baseline with each follow‐up (*t* = 1 and *t* = 2) assessment.

Results were analysed using R software (Version 4.2.0) and SPSS software Version 25.0 (IBM Corporation, Armonk, NY, USA). Graphs were performed using GraphPad Prism Version 8.0 (GraphPad Software Inc., La Jolla, CA, USA). Statistical significance was set at *p* < 0.05 for all analyses.

## 3 Results

### 3.1 Sample Demographics

Sociodemographic characteristics of the AUD and control groups at *t* = 0, *t* = 1 and *t* = 2 are summarized in Table 1. The AUD group was significantly older than the control group (*p* < 0.01), whereas sex approached the threshold for statistical significance (higher proportion of men in the AUD group was observed) (*p* = 0.05). No differences were found between groups in body mass index or total cholesterol levels. Although the AUD group tended to have lower levels of education and a higher unemployment rate compared to the control group, these differences did not reach statistical significance. Nevertheless, given the well‐established influence of education on cognitive performance [18], education was included as a covariate in all subsequent statistical models. Tobacco use did not differ significantly between groups at any timepoint. All patients received pharmacological treatment. The most frequently prescribed medication was disulfiram, followed by antidepressants and anticonvulsants (review [4]). No participants in the control group were receiving psychiatric treatment.

### 3.2 Alcohol Use and Liver Function at *t* = 0

Key findings regarding alcohol consumption in the AUD group obtained from the semi‐structured interview are presented in Table S4 (Supporting Information Section 2), including age of drinking initiation, problematic use and abstinence. Briefly, at recruitment (baseline), patients had maintained abstinence for an average of 44.94 days (SD = 16.45), and the duration of alcohol use prior to their most recent relapse averaged 37.67 weeks (SD = 19.28), indicating a chronic pattern of alcohol abuse.

Liver function was assessed using a panel of clinical markers, including ALT, AST, GGT, ALP and bilirubin levels. All patients included in the final analysis exhibited values within the normal clinical range (Table S4), confirming the absence of significant hepatic dysfunction at study entry.

### 3.3 Cognitive Function Trajectory in AUD

Cognitive performance across timepoints *t* = 0, *t* = 1 and *t* = 2 for the AUD and the control groups is summarized in Table 2. As previously established, the control group was only evaluated at *t* = 0 and t = 1 to account for a potential learning effect during repetition of tests. Because no learning effect was detected between these two assessments (paired *t*‐test, *p* > 0.05), their *t* = 2 scores were assumed to remain unchanged.

**TABLE 2:** Cognitive scores in the AUD and control groups at *t* = 0, *t* = 1 and *t* = 2.

	Cognitive domains		GCF	Visuospatial _Cognition_	^Memory^/Learning	EF
Direct scores [mean (SD)]	*t* = 0	AUD (*n* = 33)	10.55 (3.78)	4.00 (1.64)	2.76 (1.68)	3.79 (1.62)
		Control (*n* = 34)	14.85 (1.94)	5.38 (0.78)	4.79 (0.98)	4.74 (1.11)
		*p*	**< 0.001**	**< 0.001**	**< 0.001**	**< 0.01**
	*t* = 1	AUD (*n* = 29)	12.55 (2.73)	4.69 (1.17)	3.24 (1.38)	4.55 (1.24)
		Control (*n* = 32)	15.56 (1.95)	5.53 (0.76)	4.97 (0.82)	5.06 (0.91)
		*p*	**< 0.001**	**0.001**	**< 0.001**	0.07
	*t* = 2	AUD (*n* = 28)	14.11 (3.70)	4.71 (1.33)	4.50 (1.69)	4.89 (1.29)
		Control (*n* = 32)	15.56 (1.95)	5.53 (0.76)	4.97 (0.82)	5.06 (0.91)
		*p*	0.069	**0.006**	0.189	0.555

*Note:* The maximum score in GCF is 18, and cognitive impairment is established at a cut‐off point ≤ 10.5 (TEDCA test). The scores of the controls at *t* = 2 are considered identical to those obtained at *t* = 1 because there was no learning effect between *t* = 0 and *t* = 1. The significant values (*p* < 0.05, Student's *t*‐test) are denoted by bold entries in the table.

Abbreviations: AUD = alcohol use disorder, EF = executive function, GCF = general cognitive functioning, *n* = total of cases, SD = standard deviation, *t* = 0 (recruitment 1–3 months of abstinence), *t* = 1 (6‐month follow‐up), *t* = 2 (12‐month follow‐up).

At baseline, the AUD group showed significantly lower scores than controls across all cognitive domains, including GCF, Memory/Learning, Visuospatial Cognition and EF (Student's *t*‐test all *p* < 0.05). By *t* = 1, EF scores in the AUD group were no longer significantly different from controls (*p* = 0.07), and by *t* = 2, no statistical differences remained in GCF, Memory/Learning or EF (*p* > 0.05), suggesting marked cognitive recovery over time. At *t* = 0, 15 patients (45.5%) exhibited GCF deficit (based on the TEDCA GCF cut‐off point ≤ 10.5). This proportion decreased to eight patients (27.6%) at t = 1 and to only four patients (14.3%) by *t* = 2.

**FIGURE 2:**
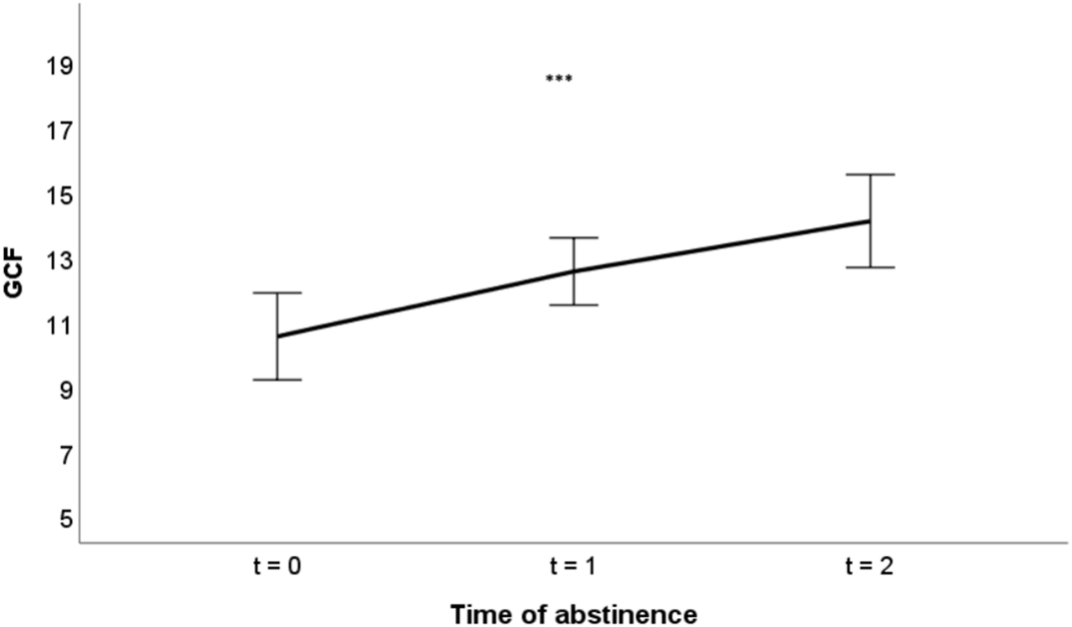
Mixed model on GCF scores with time of abstinence as factor, controlling for age, sex and education. Means and error bars (95% CI) for GCF. The horizontal axis shows the time of abstinence factor: *t* = 0 (recruitment 1–3 months of abstinence); *t* = 1 (6‐month follow‐up); *t* = 2 (12‐month follow‐up). Overall effect of time of abstinence, indicated by *** (*p* < 0.01). GCF = general cognitive function.

Mixed‐effects model (1), used to assess cognitive change (GCF) over time in the AUD group, revealed an intraclass correlation coefficient (ICC) of 0.428. This suggests that approximately 42.8% of the total variability observed in GCF can be attributed to differences between subjects, whereas the remaining 57.2% is due to variability within the same subjects over time. As previously mentioned (data analysis, methods section), two models were compared in turn, one with random intercepts (Model 1) and another that also considers random slopes (Model 2) of time on GCF. The reported model chosen was the one with random slopes, which is presented in Table 3 and illustrated in Figure 2.

**TABLE 3:** Evolution of GCF scores during time of abstinence in the AUD group.

Fixed effects	Est. (s.e.)	*p*	95% CI
Intercept	10.021 (1.522)	**< 0.001**	6.927–13.114
Time of abstinence	1.845 (0.343)	**< 0.001**	1.145–2.545
Age	−0.021 (0.079)	0.783	−0.182–0.139
Sex (male)	0.442 (1.328)	0.742	−2.275–3.158
Educ	0.441 (1.170)	0.708	−1.934–2.817

Random effects			
Residual variance	2.683 (0.727)	**< 0.001**	1.578–4.561
Intercept variance	11.919 (3.751)	**0.001**	6.432–22.087
Time slope variance	2.121 (0.999)	**0.034**	0.842–5.342
Intercept time covariance	−2.483 (1.529)	0.104	−5.480–0.515
AIC = 460.51; BIC = 483.01; deviance = 442.51			

*Note:* Mixed model with random slopes on GCF scores, with time of abstinence as factor. The results showed a significant effect of time of abstinence (Est. = 1.845), but not of the control variables (*p* > 0.05). Significant values are shown in bold.

Abbreviations: 95% CI = 95% confidence intervals, AIC = Akaike Information Criteria, BIC = Bayesian Information Criteria, Est. = estimate, s.e. = standard error.

On average, patients showed a 1.845‐point improvement (direct scores on GCF) per time interval [Est (s.e.) = 1.845 (0.343)] (Table 3). Given two intervals (*t* = 0–*t* = 1; *t* = 1–*t* = 2), this translates to an average total increase of 3.690 points from baseline to *t* = 2, with a large effect size of 0.974 (Cohen's *d*), relative to the baseline SD (*t* = 0). Consequently, there was a significant and marked improvement in GCF over time of abstinence in the AUD group.

The significance of the variance of the random slopes suggested individual differences in the extent of improvement of GCF (some subjects improve more than others). Because the standard deviation of the slopes was 1.46 and given that the distribution of the slopes was assumed to be Gaussian, about 70% of the patients showed an enhancement in their cognitive performance by 1.845 ± 1.46 points.

All model assumptions were met: normal distribution of random effects (W = 0.971, *p* = 0.508 for slopes; W = 0.976, *p* = 0.673 for intercepts), normal residuals (W = 0.987, *p* = 0.494) and homoscedasticity (*F*_LEVENE_ (2,87) = 1.374, *p* = 0.259).

### 3.4 Longitudinal Evolution of Inflammatory Biomarkers and Apolipoproteins

Plasma levels of inflammatory and lipid‐related biomarkers across timepoints are detailed in Table 4. Biomarkers were measured repeatedly in the AUD group to study longitudinal changes over abstinence, whereas control biomarker values were available only at baseline, as a reference range for inflammation and apolipoproteins in no disease conditions. Therefore, control biomarker values were not used as a repeated‐measures comparison group.

**TABLE 4:** Plasma inflammatory parameters and apolipoproteins in the AUD and control group at *t* = 0, *t* = 1 and *t* = 2.

Biomarkers		*t* = 0			*t* = 1			*t* = 2		
		AUD (*n* = 33)	Control (*N* = 34)	*p*	AUD (*n* = 29)	Control (*N* = 32)	*p*	AUD (*n* = 28)	Control (*N* = 32)	*p*
Plasma	LPS (EU/mL) [mean (SD)]	0.265 (0.199)	0.143 (0.124)	**0.004**	0.124 (0.094)	0.139 (0.124)	0.601	0.116 (0.081)	0.139 (0.124)	0.405
	LBP (μg/mL) [mean (SD)]	25.52 (12.64)	23.11 (13.27)	0.454	22.550 (9.982)	22.355 (13.205)	0.948	23.97 (10.57)	22.355 (13.205)	0.607
	APOAI (μg/mL) [mean (SD)]	69.41 (55.20)	30.68 (16.21)	**< 0.001**	43.058 (40.713)	30.200 (16.686)	0.160	42.663 (36.976)	30.200 (16.686)	0.135
	APOAII (μg/mL) [mean (SD)]	80.83 (27.46)	89.50 (31.44)	0.234	85.621 (43.661)	87.275 (30.701)	0.864	92.842 (43.378)	87.275 (30.701)	0.565
	APOB (μg/mL) [mean (SD)]	229.72 (111.50)	183.61 (120.06)	0.117	151.483 (100.310)	158.100 (81.740)	0.782	180.930 (119.650)	158.100 (81.740)	0.403
	APOCII (μg/mL) [mean (SD)]	74.14 (30.03)	72.49 (44.35)	0.861	73.724 (44.278)	71.388 (45.373)	0.840	76.400 (28.025)	71.388 (45.373)	0.620
	APOE (μg/mL) [mean (SD)]	18.09 (5.72)	13.50 (6.23)	**0.003**	14.043 (5.672)	13.232 (6.268)	0.606	13.659 (4.962)	13.232 (6.268)	0.777
	APOJ (μg/mL) [mean (SD)]	38.76 (16.51)	25.57 (13.86)	**0.001**	32.658 (18.550)	25.711 (13.903)	0.106	23.910 (15.169)	25.711 (13.903)	0.636
	APOM (mg/mL) [mean (SD)]	13.08 (6.36)	16.85 (7.30)	**0.029**	14.815 (5.953)	17.016 (7.493)	0.212	16.273 (6.672)	17.016 (7.493)	0.686

*Note:* Levels of inflammatory parameters LPS and LBP; plasma apolipoproteins APOAI, APOAII, APOB, APOCII, APOE, APOJ and APOM in the AUD and control groups at *t* = 0, *t* = 1 and *t* = 2. Grubb's test or the extreme studentized deviate (ESD) method applied. The *p‐*value from Student's *t*‐test. The significant values (*p* < 0.05) are denoted by bold entries in the table. Due to some variables exhibiting positive skewness, the results were replicated using the non‐parametric Mann–Whitney *U* test, yielding the same outcomes.

Abbreviations: SD = standard deviation, *t* = 0 (recruitment 1–3 months of abstinence), *t* = 1 (6‐month follow‐up), *t* = 2 (12‐month follow‐up).

At baseline, AUD patients exhibited significantly altered biomarker profiles compared to controls, with elevated LPS (*p* = 0.004), APOAI (*p* < 0.001), APOE (*p* = 0.003), APOJ (*p* = 0.001) and lower APOM (*p* = 0.029). By *t* = 1 and *t* = 2 months, AUD biomarker values were no longer statistically different from baseline values observed in controls, indicating biomarker normalization with sustained abstinence (Table 4).

To assess biomarker trajectories, mixed models were applied to all biomarkers (mixed models (2), review data analysis methodology section), considering time of abstinence as a factor (Level 1) and the same control variables (age, sex, education as covariates) (Level 2). All mixed models were fitted for a random intercepts model and results are shown in Table 5.

**TABLE 5:** Evolution of biomarkers during time of abstinence in the AUD group.

Fixed effects (*F* tests)				
Dependent variable	Time of abstinence (*F*‐statistic)	Sex	Education	Age
APOAI	**4.075** *	0.301	2.154	1.663
APOAII	0.746	0.277	0.025	0.143
APOB	**11.317** ***	**4.770** *	0.094	2.482
APOCII	0.075	1.088	0.519	0.063
APOE	**6.636** **	0.414	0.584	0.343
APOJ	**5.404** **	2.242	0.824	0.209
LPS	**15.801** ***	0.190	0.009	0.530
LBP	0.625	0.009	1.742	0.218
APOM	**15.493** ***	2.950	2.367	1.291

*Note:* All mixed models were fitted for a random intercepts model and utilized time of abstinence as a factor (three levels). The predictors sex and education are binary variables and age (in years) is a quantitative predictor. Significant values are shown in bold.

* *p* < 0.05.

** *p* < 0.01.

*** *p* < 0.001.

Significant effects of time of abstinence were observed (indicating a meaningful change in the values of biomarkers throughout the study) for LPS, APOAI, APOB, APOE, APOJ and APOM (Table 5):

- LPS levels decreased significantly from *t* = 0 to *t* = 1 (adjusted difference = 0.144, *p* < 0.001, *d* = 0.723) and from *t* = 0 to *t* = 2 (adjusted difference = 0.152, *p* < 0.001, *d* = 0.763).
- APOAI decreased over time: *t* = 0–*t* = 1 (adjusted difference of 29.155, *p* = 0.021, *d* = 0.544) and *t* = 0–*t* = 2 (adjusted difference of 31.535, *p* = 0.019, *d* = 0.588).
- APOB showed reductions from *t* = 0 to *t* = 1 (adjusted difference = 83.871, *p* < 0.001, *d* = 0.752) and *t* = 0 to *t* = 2 (adjusted difference = 53.352, *p* = 0.006, *d* = 0.478).
- APOE decreased from *t* = 1 to *t* = 2 (adjusted difference = 4.012, *p* = 0.005, *d* = 0.701) and *t* = 0 to *t* = 2 (adjusted difference = 4.559, *p* = 0.003, *d* = 0.797).
- APOJ showed a significant drop from *t* = 0 to *t* = 2 (adjusted difference = 15.094, *p* = 0.003, *d* = 0.914).
- APOM, in contrast, increased over time: *t* = 0–*t* = 1 (adjusted difference = −2.851, *p* = 0.001, *d* = 0.448) and *t* = 0–*t* = 2 (adjusted difference = −4.344, *p* < 0.001, *d* = 0.683).

All model assumptions were met, including linearity, independence, homoscedasticity and normality of Level 1 residuals and random intercepts. No issues of collinearity or model misspecification were detected (data not shown).

### 3.5 Association Between Biomarker Changes and Cognitive Improvements in AUD

#### 3.5.1 Multivariable Mixed‐Effects Model Predicting GCF

To examine how changes in cognition (GCF) related to changes in biomarkers throughout the same abstinence process (*t* = 0, *t* = 1 and *t* = 2) in the AUD group, we applied a mixed model analysis (3). APOAI was excluded due to its non‐significant contribution in preliminary models (data not shown) with 21.2% missing data in multiplex quantification (under detection levels). This exclusion aimed to preserve model stability without resorting to imputation. The final model included biomarkers that had shown significant changes over time in Model 2 (LPS, APOB, APOE, APOJ and APOM), with abstinence time treated as a Level 1 predictor. Sex, age and education were entered as Level 2 covariates, along with subject‐level aggregated means of the biomarkers, to estimate between‐person biomarker effects (see Section 2). The adjusted mixed model featured random intercepts, but the variances of the slopes for the Level 1 predictor effects were excluded due to their lack of significant improvement in the model.

The results of all these analyses are summarized in Table 6 and briefly explained here:

- Abstinence time was a significant positive predictor of GCF (*p* = 0.019). At each assessment (*t* = 0, *t* = 1 and *t* = 2), GCF scores increased by 1.262 points on average in patients, controlling for biomarker effects [Est (s.e.) = 1.262 (0.516)].
- The within‐person component of LPS had a significant negative association with GCF (*p* = 0.015). Specifically, when participants showed lower‐than‐usual LPS levels relative to their own average, they tended to show higher GCF scores [Est (s.e.) = −6.787 (2.670)]. This suggests that intraindividual decreases in LPS were associated with better cognitive functioning during abstinence.
- No apolipoprotein component reached statistical significance in the final model. APOM within‐person and APOJ between‐person showed borderline associations, but these findings were not robust in sensitivity analyses and should be interpreted as exploratory.

Regarding the model assumptions, Shapiro–Wilk normality test for random intercepts showed conformity to normality (W = 0.958, *p* = 0.234) as well as for the Level 1 residuals (W = 0.984, *p* = 0.431). Additionally, the evaluation of homoscedasticity, based on the variances of residuals across the three timepoints of abstinence, also showed no issues (*F*_LEVENE_ (2,74) = 0.410, *p* = 0.665).

**TABLE 6:** Mixed‐effects model predicting GCF from time of abstinence, within‐person biomarker deviations, between‐person biomarker means and covariates in the AUD group.

Fixed effects	Est. (s.e.)	*p*	95% CI
Intercept	8.072 (5.378)	0.149	−3.165–19.310
Sex	0.875 (1.234)	0.486	−1.681–3.431
Age	−0.032 (0.070)	0.651	−0.177–0.113
Education	0.874 (1.064)	0.420	−1.337–3.086
Time of abstinence	1.262 (0.516)	0.019*	0.221–2.303
APOB (*within*)	0.008 (0.005)	0.162	−0.003–0.019
APOE (*within*)	−0.022 (0.086)	0.801	−0.195–0.151
APOJ (*within*)	0.020 (0.019)	0.293	−0.018–0.059
APOM (*within*)	0.221 (0.117)	0.066	−0.015–0.458
LPS (*within*)	−6.787 (2.670)	0.015*	−12.188 to −1.385
APOB (*between*)	0.001 (0.006)	0.883	−0.011–0.013
APOE (*between*)	−0.153 (0.141)	0.290	−0.444–0.139
APOJ (*between*)	0.094 (0.046)	0.051	−0.0003–0.189
APOM (*between*)	0.111 (0.117)	0.351	−0.129–0.350
LPS (*between*)	0.529 (5.109)	0.919	−10.154–11.212

Random effects			
Residual variance	4.879 (1.150)	< 0.001***	3.073–7.745
Intercept variance	4.638 (2.210)	0.036*	1.822–11.802
AIC = 397.373; BIC = 401.628; deviance = 393.373			

*Note:* Mixed‐effects model with random intercepts for participants. Time‐varying biomarkers were decomposed into within‐person (within) deviations and person‐level means (between). Within‐person biomarker components represent deviations from each participant's own mean across repeated assessments, whereas person‐level means estimate between‐person differences in average biomarker levels.

Abbreviations: 95% CI = 95% confidence intervals, AIC = Akaike Information Criteria, APOB = apolipoprotein B, APOE = apolipoprotein E, APOJ = apolipoprotein J, APOM = apolipoprotein M, BIC = Bayesian Information Criteria, Est. = estimate, GCF = general cognitive functioning, LPS = lipopolysaccharide, s.e. = standard error.

* *p* < 0.05.

** *p* < 0.01.

*** *p* < 0.001.

#### 3.5.2 Correlation Matrices and Sensitivity Analyses Within‐ and Between‐Subject and Interval‐Specific Reduced Mixed‐Effects Models

Sensitivity analyses were conducted to address model complexity and the potential influence of multiple biomarkers. Correlation matrices did not suggest severe multicollinearity among biomarkers (Tables S5 and S6). Reduced mixed‐effects models including LPS and one apolipoprotein at a time (Table S7) showed that within‐person LPS remained negatively associated with GCF across all model specifications, whereas apolipoproteins did not show robust independent associations after accounting for LPS, abstinence time and covariates. Interval‐specific models comparing *t* = 0–*t* = 1 and *t* = 0–*t* = 2 separately confirmed that within‐person LPS remained negatively associated with GCF in both comparisons, whereas the time effect was evident mainly in the *t* = 0–*t* = 2 comparison (Table S8). Therefore, the LPS finding appears comparatively robust, whereas apolipoprotein results should be interpreted as exploratory.

## 4 Discussion

The main results shown in this study are as follows: (a) AUD patients show GCF impairment and alterations in inflammatory (LPS) and apolipoproteins (APOAI, APOE, APOJ and APOM) during early abstinence (*t* = 0) that evolve during prolonged abstinence, after controlling for important covariables. Specifically, GCF showed a trajectory of improvement during the dishabituation phase, reaching cognitive abilities similar to controls at 12 months of abstinence. Significant differences between biomarker levels in AUD patients and (normal range) levels in controls were no longer found in the follow‐up (*t* = 1 and *t* = 2). (b) The effect of time of abstinence (from *t* = 0 to *t* = 2) and normalization of plasma LPS levels (from *t* = 0 to *t* = 1/*t* = 2) remained consistently associated with GCF improvement, according to reduced mixed‐effects models. In contrast, apolipoproteins did not show robust independent associations with GCF after accounting for LPS and covariates.

### 4.1 Cognitive Recovery in AUD

GCF improved significantly over the 12‐month follow‐up, supporting previous findings that cognitive deficits in AUD may be at least partially reversible through sustained abstinence [19, 20, 21, 22, 23]. In our study, abstinence time remained an independent predictor of cognitive improvement, with GCF increasing by 1.262 points per assessment. Therefore, abstinence per se may exert a robust and independent contribution to cognitive recovery. It could be said that improvement is observed at intervals of 1–3, 6 and 12 months, according to previous studies [24, 25, 26, 27], but, interestingly, recovery trajectories varied across different cognitive domains.

EF showed the most prominent recovery, with differences from controls no longer significant by 6 months of follow‐up. This is in line with prior evidence suggesting that EF‐related processes, including working memory, divided attention and inhibitory control, may recover relatively early during abstinence [3, 23, 28]. In a controlled 18‐day detoxification study, working memory improved, whereas inhibition did not, and trajectories varied across subjects, patterns that mirror the heterogeneity we observed and that underscore the need for targeted cognitive follow‐up [29].

Improvements in memory and learning were also observed, particularly at 12 months of follow‐up. These results corroborate findings from studies reporting partial restoration of episodic memory within the first 6 months of abstinence [26], although persistent hippocampal dysfunction has been noted in some studies [30, 31].

Visuospatial cognition showed a more attenuated recovery profile. Despite initial impairment at baseline, improvements over time were more modest, a pattern that aligns with prior evidence indicating reduced plasticity in visuospatial functions following chronic alcohol exposure [32, 33]. Nonetheless, partial gains have been described in some subjects [34, 35], and our findings are consistent with this trajectory.

Finally, the heterogeneity of recovery trajectories across domains aligns with prior findings suggesting that cognitive recovery in AUD may depend on numerous factors [36] and suggests the need for individualized cognitive monitoring and intervention strategies [29, 37].

### 4.2 Evolution of Inflammatory Biomarkers and Apolipoproteins During Abstinence and Associations With Cognition in AUD

Regarding apolipoproteins and AUD, longitudinal studies assessing changes in plasma levels during alcohol abstinence are, to date, non‐existent. Nevertheless, longitudinal assessments have been conducted in other diseases and under different conditions [38]. Very limited studies linked plasma apolipoproteins with inflammation and cognition in the context of AUD [4], and, to our knowledge, this is the first study including longitudinal evolution of apolipoproteins, inflammatory markers and cognition during abstinence, as mentioned previously.

Our AUD cohort showed higher LPS, APOAI, APOE, APOJ and lower APOM than controls at *t* = 0, with no group differences in LBP, APOAII, APOCII and only a non‐significant trend for APOB (which, however, fluctuates across the months of follow‐up in AUD patients, despite showing no difference with control levels). By 6 months of follow‐up, all markers converged to control‐like levels and remained stable thereafter, indicating that the largest biological change occurs early in abstinence.

In our study, plasma LPS was elevated at baseline but declined by 6 and 12 months, reaching control values. This may be consistent with partial restoration of gut barrier integrity and reduced endotoxemia during recovery. Higher circulating LPS at *t* = 0 plausibly contributed to cognitive deficits in AUD, in line with prior evidence suggesting that peripheral inflammation and LPS perturbs brain homoeostasis and behaviour and induces cognitive impairments [39, 40], as suggested also for alcohol binge drinking [41].

In the context of alcohol consumption, there are several ways in which systemic immune activation can promote neuroinflammation and trigger cognitive decline [6, 7, 42]. Alcohol abuse leads to increased intestinal permeability, allowing bacterial products such as LPS to enter the systemic circulation, activating innate immune receptors and initiating a pro‐inflammatory cascade. Peripheral pro‐inflammatory cytokines influence the enteric nervous system via the vagus nerve, activating the HPA axis and conveying cytokine signals to the brain. They can also reach the brain or signal through circumventricular organs or the blood–brain barrier (through dysfunction/opening or through the activation of specific signalling mechanisms, such as activation of perivascular cell receptors and prostaglandin receptors), thereby affecting the brain. For example, sustained brain inflammation in response to circulating endotoxin requires brain TLR4, which can be found in microglia, endothelium, perivascular macrophages, meninges or circumventricular organs [42]. This suggests that the long‐term effect of LPS on the brain may be mediated in part by the activation of these cells that induce brain cytokine production. Alternatively, certain components of *Escherichia coli* LPS can infiltrate the rat brain under physiological conditions bound apolipoproteins [43], being found in tanycyte‐like cells at the blood–cerebrospinal fluid interface, as well as in ependymal cells of circumventricular organs and even in astrocytes of the medulla oblongata [43]. Astrocytes and tanycytes in the brain's circumventricular organs are known to be crucial for initiating LPS‐induced inflammatory responses via TLR4 [44]. Infiltration of LPS to brain areas could be happening after alcohol abuse, due to alterations in the blood–brain barrier [45]. Indeed, infiltration of LPS components has been described in the frontal cortex of animals after alcohol binges, aggregated to sex‐dependent apolipoproteins, with consequences in neuroinflammation [11].

Whatever the possible mechanism (sympathetic/vagal signalling, receptor activation at brain interfaces or molecular infiltration), in the current longitudinal study we observed that within‐person change in LPS predicted change in cognition. Specifically, a one‐unit decrease in LPS was associated with a 6.79‐point increase in TEDCA‐derived GCF between follow‐ups. This positions LPS as a candidate biomarker for monitoring cognitive risk and recovery during treatment.

Similarly, apolipoproteins normalize their levels during abstinence. Thus, APOAI, APOE and APOJ decreased, whereas APOM increased over the period of follow‐up. It is interesting to note that APOM is integrated within HDL molecules as well as APOAI, which is majority [46, 47, 48], and both APOAI and APOM are altered in plasma in opposite directions during AUD abstinence. Their divergence suggests that distinct HDL‐related processes are occurring during abstinence: an inflammation‐loaded state indexed by elevated APOAI and decreased APOM early on, and a progressively ‘protective’ HDL phenotype indexed by regulation of APOAI plasma levels and especially by the rising of plasma APOM as systemic inflammation decreases. This pattern integrates well with our prior clinical work showing that, at baseline, plasma APOAI was higher and APOM was lower in AUD patients, and APOAI correlated positively with LPS while APOM correlated negatively [4]. Despite APOM consistently increasing across follow‐up, according to its anti‐inflammatory and LPS detoxification/neutralization properties [49, 50]_,_ its association with GCF did not reach statistical significance and was not robust in sensitivity analyses. Similarly, the normalization of other apolipoproteins during abstinence did not predict the cognitive improvement in the final model, which contrasts with a proposed role for APOAI in memory impairment in alcohol‐dependent mice and AUD patients [16] (but see also limitations of this study), so any changes should be interpreted as exploratory.

### 4.3 Limitations

Longitudinal studies with AUD patients are difficult to perform due to loss of subjects along the study, economic constraints and relapses across the study. Although the current study has the strength of any longitudinal study, plus the combination of biological and cognitive measures, these types of studies are not exempt from limitations, which we analyse here: (1) The sample size and potential attrition across 12 months may limit power to detect small or domain‐specific cognitive effects as well as identify possible sex‐dependent effects. In addition, the sample size was modest relative to the number of biomarkers and covariates included in the final multivariable model. Although mixed‐effects models are appropriate for repeated‐measures data and incomplete follow‐up, the ratio of predictors to participants may increase the risk of unstable estimates, reduced power and overfitting. We therefore conducted correlation matrix, reduced‐model sensitivity analyses and interval‐specific sensitivity analyses models comparing *t* = 0, *t* = 1 and *t* = 0 and *t* = 2. Although the correlation matrices and reduced sensitivity analyses did not suggest severe multicollinearity and supported the consistency of the within‐person LPS–GCF association across alternative model specifications, the number of biomarkers examined remains large relative to the modest sample size. Therefore, the multivariable biomarker findings should be interpreted cautiously. The most consistent results across sensitivity analyses were the positive association between time of abstinence and GCF and the negative association between within‐person LPS and GCF. In contrast, apolipoprotein associations were less stable and should be considered exploratory pending replication in larger longitudinal cohorts. (2) The control group was significantly younger than the AUD group. Although age was included as a covariate in the mixed‐effects models, residual age‐related confounding cannot be fully excluded, particularly for baseline between‐group comparisons of cognitive performance and plasma biomarkers. Therefore, comparisons with controls should be interpreted as reference comparisons, whereas the strongest inference of the study derives from within‐person longitudinal change in the AUD group. (3) Biomarkers were not repeatedly measured in the control group. Therefore, AUD biomarker values at follow‐up were compared with baseline control values as a reference for normal values. Although not expected, we cannot determine time‐related fluctuations in these biomarkers in a 6‐ or 12‐month period, which constitutes a possible limitation. (4) Peripheral biomarkers are a good tool to use in the clinical setting, but we do not have correspondence for central biomarkers, so what happens within the brain remains speculative. (5) The measurement of multiple apolipoproteins from a single plasma sample may carry methodological inaccuracies because the sample dilution fits most of the proteins, but it can be some others out of range. This was the only case for APOAI in this study, in which we lost around 21.2% of the sample, which made it difficult to obtain significant results in the statistical models. (6) We may have missed rapid biomarker fluctuations during very early abstinence, out of the *t* = 0, *t* = 1 and *t* = 2 points of sampling, so normalization of biomarkers would have had a more significant impact taking into account a very early inflammatory and cognitive status. (7) Although we adjusted for key covariates (age, sex and education), we cannot discard residual confounding factors such as lifestyle factors (diet and smoking), common psychiatric symptoms or the effect of medication and psychological therapy to maintain the abstinence along the alcohol programme.

### 4.4 Conclusion

We observed that, in AUD patients included in an ambulatory dishabituation programme, cognitive function improved over 12 months and abstinence duration remained independently associated with this recovery. Systemic inflammation receded, reflected by a decline in plasma LPS levels during follow‐up, and within‐person reductions in LPS were associated with better GCF, supporting a potential link between reduced systemic inflammation and cognitive improvement. The apolipoproteins that were upregulated at baseline in AUD patients (APOAI, APOE and APOJ) decreased to control levels over abstinence (*t* = 1), and the opposite pattern was observed with APOM (the initial downregulation was normalized at *t* = 1). Nevertheless, these apolipoproteins did not independently predict cognitive change in the final model, so their role should be interpreted as part of a broader immune–lipid remodelling process rather than as direct predictors of cognitive recovery. Together, these findings support further studies with larger samples and repeated biomarker assessment in age‐matched controls to determine whether LPS and selected apolipoproteins may serve as markers of recovery in AUD.

## Author Contributions

**B.E.:** conceptualization, investigation, formal analysis, writing – original draft. **R.O**.: methodology, formal analysis. **F.A.:** methodology, resources. **L.O.:** conceptualization, supervision, project administration, funding acquisition, writing – review and editing.

## Funding

This work was supported by the Ministerio de Ciencia, Innovación y Universidades (PID‐2021‐127256OB‐I00) and the Instituto de Salud Carlos III (RD24/0003/0010).

## Ethics Statement

This study was part of a research project approved by the research ethics committee of the Hospital 12 de Octubre, Madrid (CEIm: 19/002) and conformed to the provisions of the Declaration of Helsinki. All participants signed informed consent to participate in the study, and anonymity was preserved.

## Conflicts of Interest

The authors declare no conflicts of interest. The materials for this study have been supported by MINECO‐FEDER Funds (PID‐2021‐127256OB‐I00 to L.O.) and RIAPAd/ISCIII (RD24/0003/0010 to L.O.).

## Supporting information


**Table S1:** Examples of alcohol consumption questions used in the semi‐structured interview for AUD and control groups.
**Table S2:** Battery of neuropsychological subtests included in TEDCA.
**Table S3:** BDI‐II and BAI test interpretation.
**Table S4:** Alcohol abuse outcomes and liver function in the AUD group.
**Table S5:** Pearson correlations among within‐person biomarker components.
**Table S6:** Pearson correlations among between‐person biomarker components.
**Table S7:** Reduced mixed‐effects models including LPS and one apolipoprotein at a time.
**Table S8:** Interval‐specific reduced mixed‐effects models comparing *t* = 0–*t* = 1 and *t* = 0–*t* = 2.

## Data Availability

The data that support the findings of this study are available on request from the corresponding author. The data are not publicly available due to privacy or ethical restrictions.
