# Supplementary material for: Longitudinal Cognitive Evolution in Alcohol Use Disorder Patients: Role of Inflammation, Time of Abstinence and Apolipoprotein Profile
Source: Addict Biol. 2026 Jul 1;31(7):e70174. doi: 10.1111/adb.70174 (PMC13322783; doi:10.1111/adb.70174)
Supplement: Supplementary file 1 — Table S1: Examples of alcohol consumption questions used in the semi‐structured interview for AUD and control groups. Table S2: Battery of neuropsychological subtests included in TEDCA. Table S3: BDI‐II and BAI test interpretation. Table S4: Alcohol abuse outcomes and liver function in the AUD group. Table S5: Pearson correlations among within‐person biomarker components. Table S6: Pearson correlations among between‐person biomarker components. Table S7: Reduced mixed‐effects models including LPS and one apolipoprotein at a time. Table S8: Interval‐specific reduced mixed‐effects models comparing t = 0–t = 1 and t = 0–t = 2. [file ADB-31-e70174-s001.docx]

**SUPPORTING INFORMATION**

1. **SUPPORTING METHODOLOGY**

**1.1. Participant Recruitment and Treatment Program AUD group**

AUD Group

Participants in the AUD group were recruited from the structured ‘*Alcohol Programme’* at Hospital Universitario 12 de Octubre (Madrid, Spain). This program has a well-established protocol and has been in clinical practice for over two decades for the treatment of alcohol-dependent patients. The program is of considerable duration, with patients being monitored multiple times per week for a period of approximately two years.

The Alcohol Programme consists of two sequential phases:

1. Detoxification Phase: This initial stage is reserved for patients presenting with acute withdrawal symptoms, requiring inpatient stabilization and medical supervision.
2. Dishabituation Phase: Extending over approximately two years, this phase involves a comprehensive psychosocial intervention combining individual and group-based therapy. Group sessions are organized into four sub-phases:

- Phase 1: Psychoeducation (12 sessions)
- Phase 2: Relapse Prevention (16 sessions)
- Phase 3: Social Skills Training (12 sessions)
- Phase 4: Discharge Preparation (variable duration)

Individual consultations are carried out with healthcare practitioners (psychologists, psychiatrists, and nurses) contingent upon the program and requirements of each individual. When appropriate, patients are referred to additional specialty services such as gastroenterology or internal medicine for further evaluation and management.

Control Group

Healthy control participants were recruited from the general population of Madrid, Spain, through convenience sampling by the research team. Recruitment was conducted via word of mouth and included hospital staff, university professionals, relatives, friends, and neighbors of the scientific team. Potential participants were contacted through email, telephone, or in person.

All participants—both patients and controls—were fully informed about the nature and purpose of the study and provided written informed consent prior to participation.

**1.2 Ensuring Compliance with Inclusion/Exclusion Criteria**

Inclusion Criteria Monitoring

To ensure adherence to abstinence at study entry, patients in the AUD group underwent a highly structured monitoring protocol during the initial phase of the Alcohol Programme. During the first month, participants received daily visits with daily breathalyzer testing to objectively verify abstinence. Abstinence was monitored through exhaled breath controls (Dräger Alcotest 6810 device (AlcoDigital, UK) during hospital visits. We ensured that all patients-maintained alcohol abstinence for a minimum of four weeks prior to baseline cognitive and biomarker assessment.

From the second to the fourth month, clinical visits became progressively less frequent:

- Second month: three times per week
- Third month: twice per week
- Fourth month onwards: once per week

In addition to breath alcohol testing, self-reported abstinence was systematically collected and considered throughout follow-up.

Exclusion Criteria

- Diagnosis of AUD in the patient group was established using the DSM-5 criteria.^1^
- For the control group, risky alcohol consumption was screened using the 'Alcohol Use Disorders Identification Test' (AUDIT),^2^ a short questionnaire and self-report screening instrument that examines risk of alcohol drinking, symptoms of dependence and harmful alcohol consumption. If detected risky consumption, the control participant was excluded from the study.
- Psychiatric comorbidity was evaluated using the Mini International Neuropsychiatric Interview (MINI), Spanish version,^3^ which covers the main DSM-IV Axis I disorders. If detected, the participant was excluded from the study.
- The Beck Depression Inventory-II (BDI-II)^4,5^ and Beck Anxiety Inventory (BAI)^6^ assess depressive or anxiety symptoms (respectively) according to their severity and were administered as additional control measures to ensure compliance with exclusion criteria.
- Additionally, a semi-structured clinical interview was conducted for both AUD Gand control groups to gather information on alcohol use history (alcohol abuse variables) and use or abuse of other drugs. Examples of the questions used are presented in Table S1.

**Table S1.** Examples of alcohol consumption questions used in the semi-structured interview for AUD and control groups

| AUD group | (A) What was the age of first contact? |
| --- | --- |
|  | (B) What is the current abstinence time in weeks/months? |
|  | (C) How many bottles, cans or glasses of beer, wine, cocktails (for instance: drinks containing liquor) did you consume per day? |
|  | (D) What was the preferred means of consumption? |
| Control group | (A) What is the current abstinence time in days? |
|  | (B) How many bottles, cans or glasses of beer, wine, cocktails (i.e. drinks containing liquor) did you consume per month? |

*Note*: Harmful drinking patterns in controls were evaluated based on quantity consumed over the prior months and the self-reported duration of current abstinence in days.

**1.3 Test of Detection of Cognitive Impairment in Alcoholism and Clinical Assessment**

The "Test of detection of cognitive impairment in alcoholism" (TEDCA)^7^ was administered to all participants by a licensed psychologist with extensive training and experience in neuropsychological testing. In the AUD group, the assessment was conducted only after participants had maintained a minimum of four weeks of verified abstinence, ensuring the absence of acute withdrawal symptoms or residual intoxication that could confound cognitive performance.

TEDCA is a screening tool specifically validated for alcohol-dependent subjects, with strong psychometric properties. TEDCA provides a snapshot of cognitive functioning at the time of application including a compendium of seven tests that assess GCF and three cognitive functions (Table S2):

- Visuospatial Cognition
- Memory/Learning
- Executive Function.

The final score meaning general intelligence or GCF results from the sum of the three previous ones. The maximum possible score on TEDCA is 18 points, and a total score of ≤ 10.5 is considered indicative of clinically significant cognitive impairment. This cut-off point has been validated in previous studies to distinguish cognitively impaired individuals from healthy controls with sensitivity and specificity.

Control group participants were only evaluated at baseline (t=0) and at 6 months follow-up (t=1). As no significant differences were observed between these two timepoints (confirmed via paired *t*-test; *p* > 0.05), the t=2 (12 months follow-up) scores were imputed as equal to t=1. This assumption is commonly accepted in longitudinal studies when no meaningful change is expected in a low-risk population and avoids overfitting models with unnecessary parameters. Moreover, controls showed no clinical or pharmacological changes across follow-up, further supporting the assumption of stability.

**Table S2.** Battery of Neuropsychological Subtests included in TEDCA

| **Cognitive domain** | **Domain description** | **Neuropsychological test** |
| --- | --- | --- |
| Visuospatial cognition | Visuo-perceptive, visuo-spatial and visuoconstruction abilities | Rey Complex Figure Test (Copy Condition)^8^ |
|  |  | Bender Visuo-Motor Gestaltic Test^9^ |
| Memory/learning | Ability to store information in the short and long term | Direct and Inverse Digits, Numbers and Letters, and Learning List from the Wechsler Memory Scale (WMS-III)^10^ |
| EF | Superior cognitive ability. Alternate/divided attention, working memory, planning, organization, problems resolution, abstraction and response inhibition | Trail Making Test B^11^ |
|  |  | Similarities Test from the Wechsler Adult Intelligence Scale (WAIS-IV)^12^ |
|  |  | A Go-No Go task |

The Beck Depression Inventory-II (BDI-II)^4,5^ and the Beck Anxiety Inventory (BAI)^6^ were used to evaluate the severity of depressive or anxiety symptoms, respectively, in the AUD group, despite the absence of formal psychiatric diagnoses. The same tests were used in the control group.

**Table S3**. BDI-II and BAI test interpretation

| **Scores and associated Interpretation** | |
| --- | --- |
| **BDI-II** | **BAI** |
| 0-13: minimal depression | 0-9: minimal anxiety |
| 14-19: mild depression | 10-15: mild anxiety |
| 20-28: moderate depression | 16-25: moderate anxiety |
| 29-63: severe depression | 26-63: severe anxiety |

*Note.* The BDI-II and BAI scores range from 0 to 63. Abbreviations: [BDI=Beck Depression Inventory-II] [BAI=Beck Anxiety Inventory].

**1. 4. Sample Processing**

After collecting, venous blood samples were centrifuged at 1800 rpm for 10 minutes at 4°C to separate plasma. The resulting plasma was carefully aliquoted into labeled vials and stored at –80°C until biomarker analysis. All assays were performed on plasma samples following standardized immunoassay protocols.

**1. 5. Human Enzyme-Linked Immunosorbent Assays for biomarkers**

Plasma Apolipoproteins (APOAI, APOAII, APOB, APOCII, APOE) levels

Plasma concentrations of APOAI, APOAII, APOB, APOCII, and APOE were measured using MAGPIX® multiplex immunoassay technology (xMap; Luminex Corporation). The analysis employed the Human Apolipoprotein Magnetic Bead Panel kit (ProcartaPlex^TM^ Human Apolipoprotein Panel 5-Plex; Catalog Number: EPX050-15818-901), targeting the five apolipoproteins of interest.

Prior to analysis, plasma samples stored in -80^◦^C were thawed on ice. Samples were diluted 1:20.000, mixed thoroughly and loaded onto 96-well plates (25 μL). All values obtained from the assay fell within the acceptable range according to recommendations from the manufacturer's instruction. Sensitivity was <15%. Final protein concentrations were calculated using the Procarta Plex^TM^ Analysis App on ThermoFisher Connect (https://apps.thermofisher.com/apps/procartaplex) and reported in μg/mL.

Plasma LPS levels

Plasma LPS levels were determined using commercially available kit following the manufacturer’s instructions (Product #: Limulus Amebocyte Lysate (LAL) Chromogenic Endpoint Assay, HIT302) (HycultBiotech, Uden, The Netherlands).

The assay is based on the LAL response of the horseshoe crab (Limulus polyphemus), in which bacterial endotoxins activate a protease cascade that produces turbidity and gelation. In this assay, the enzymatic reaction generates a yellow colour, whose optical density is measured at 450 nm in a spectrophotometer (Molecular Devices®; Ramsey). Results (endotoxin units per mL (EU/mL)) were reported in pg/mL.

Plasma LBP levels

Plasma levels of LBP were measured using a commercially available ELISA kit (Product #: Human LBP, HK315-01) (HycultBiotech, Uden, The Netherlands), following the manufacture’s protocol. Samples were diluted 1:1000 and incubated in microtiter wells pre-coated with anti-human LBP antibodies. The optical density was measured at 450 nm with a spectrophotometer (Molecular Devices®; Ramsey). This assay had a measurable range of 4.4 to 50 ng/mL, with an intra-assay coefficient of variation below 5%. Final concentrations were reported in μg/ mL.

Plasma APOM and APOJ levels

Plasma levels of APOM and APOJ were quantified using commercial ELISA kits according to the manufacturer's instructions (APOM: Human Apolipoprotein M (ApoM) ELISA kit (CUSABIO, Houston, USA), catalogue number: CSB-EL001947HU; and APOJ: RayBio® Human Clusterin Elisa kit; reference: ELH-Clusterin (RayBiotech). Plasma samples were 1:2000 (APOM) and 1:50000 (APOJ) diluted. The optical density was detected using a microplate reader set to 450 nm and both APOM and APOJ concentrations were determined from the standard curve. The minimum detectable concentration for APOM (sensitivity) was <0.39 ng/mL (detection range: 1.56 ng/mL to 100ng/mL; and intra-assay coefficient of variation <8%). For APOJ, the sensitivity was 15 pg/mL and intra-assay coefficient of variation was less than 10%. Final concentrations were reported as mg/mL for APOM and μg/mL for APOJ.

**1.6. Mixed-effects Models.**

To analyze longitudinal changes and predictive relationships across timepoints, a series of linear mixed-effects models were applied:

1. Model 1 examined changes in GCF over time (t=0, t=1, t=2) within the AUD group. Time in abstinence was considered a quantitative numerical variable, rather than a factor, given the established linear relationship between the GCF variable and abstinence time. Abstinence time was considered a predictor (level 1) and sex, age, and education were considered covariates (level 2).
2. Model 2 evaluated longitudinal changes in plasma biomarkers (LPS, LBP, and apolipoproteins) across the abstinence period. Here, abstinence time was considered a factor, as a linear relationship between each biomarker and abstinence time did not exist in all cases. Abstinence time was considered a predictor (level 1), and the covariates were sex, age, and education (level 2).
3. Model 3 assessed whether changes in biomarkers that showed significant variation in Model 2 (LPS, APOAI, APOB, APOE, APOJ and APOM) were associated with cognitive changes (GCF) over the same abstinence process. Abstinence time and the previously significant biomarkers were the predictors (level 1), while sex, age, and education were covariates (level 2). To control contextual effects, all level 1 predictors (except abstinence time, which lacks inter-individual variance) were accompanied by their corresponding group-level aggregates at level 2, alongside the demographic covariates (level 2: covariates + the aggregated biomarkers).

To determine the optimal random structure of each model, two specifications were compared: 1. a random intercept model (assuming homogeneous change across subjects, e.g., GCF in mixed model 1 is constant for all subjects) and 2. a random slope model (allowing individual variability in trajectories over time). To compare the random structure of these two models, model selection indices such as the likelihood ratio, Akaike Information Criterion (AIC), and Bayesian Information Criterion (BIC) were used.

It was assumed that the amount of unexplained variation in the results (residual variances) was the same at the three abstinence points (t=0, t=1, t=2). Similarly, it was assumed that the residuals, as well as the intercepts and slopes, followed a bivariate normal distribution. Restricted maximum likelihood (REML) was used as the estimation method for the three mixed models. The normality of the residuals and random effects was verified with the Shapiro-Wilks test, and the homoscedasticity of the residuals was tested with Levene's test. The lme4 library in R^13^ was used to fit the mixed models, while the *pbkrtest* library^14^ was used to estimate statistical significance, adjusting the degrees of freedom using the Kenward-Roger method (for an explanation, see^15^). SPSS 25.0 was used for the *Wald* test of the variance of random effects.

To distinguish intraindividual biomarker changes from stable between-participant differences, time-varying biomarkers included in the mixed-effects models were decomposed into within-person and between-person components. Within-person components were computed by person-mean centering each biomarker across repeated assessments, whereas between-person components corresponded to each participant’s average biomarker level. Both components were included in the multivariable model predicting GCF.

Sensitivity analyses were conducted to address model complexity and the potential influence of multiple biomarkers. Correlation matrices did not suggest severe multicollinearity among biomarkers.

**SUPPORTING RESULTS**

**2.1 Alcohol abuse variables and liver parameters**

**Table S4.** Alcohol Abuse Outcomes and Liver Function in the AUD Group

| Alcohol abuse variables [mean, (SD)] N = 33 | Duration of alcohol abuse since last relapse (weeks) | 37.67 (19.28) |
| --- | --- | --- |
|  | Age of problematic drinking initiation (years old) | 15.12 (3.72) |
|  | Age of problematic drinking initiation (years old) | 29.91 (11.22) |
|  | Duration of abstinence since last consumption at recruitment (days) | 44.94 (16.45) |
|  | SDU per month | 24.88 (13.02) |
| Liver status markers [mean, (SD)] N = 30–33 | ALT (U/L) (n=33) | 32.42 (26.36) |
|  | (AST (U/L) (n = 33) | 30.09 (26.24) |
|  | GGT (U/L) (n = 32) | 54.78 (59.05) |
|  | ALP (U/L) (n = 30) | 81.03 (23.28) |
|  | Bilirubin (mg/dL) (n = 31) | 0.57 (0.37) |

*Note*. The *duration of alcohol abuse* variable refers to the length of time the patient has been consuming alcohol since their most recent relapse. *Standard drinking units* (SDU) are calculated as: volume of alcohol in liters x percentage of alcohol contained in the beverage x 0.8 (because 1 mL of alcohol contains 0.785 g of alcohol). Liver parameters: the European reference values (inner and upper limits) for each parameter (IU/L) are as follows: ALT: 5–45; AST: 5–33; GGT: 8–61; ALP: 40–130; bilirubin: 0.2–1.0. Data (n = 30–33) are expressed as mean (SD). Abbreviations: ALP, alkaline phosphatase; ALT, alanine aminotransferase; AST, aspartate aminotransferase; AUD, alcohol use disorder; GGT, gamma-glutamyl transferase; SDU, standard drinking units.

**2.2 Correlation matrix among biomarkers**

To assess potential collinearity among biomarkers, we examined correlation matrices after decomposing time-varying biomarkers into within-person deviations and between-person components. This approach allowed us to distinguish intraindividual change from stable between-participant differences. Within-person components were computed as deviations from each participant’s own mean across repeated assessments, whereas between-person components corresponded to each participant’s average biomarker level.

The correlation matrix (Table S5 and Table S6) did not suggest severe multicollinearity. The highest correlation among within-person biomarker components was moderate (APOB (within)–APOE (within), r = 0.527), and the highest correlation among between-person biomarker means was observed between APOJ (mean) and APOM (mean) (r = -0.661). Although this latter association was moderate-to-high, it remained below commonly used thresholds for severe pairwise collinearity. Nevertheless, given the modest sample size, we now interpret the multivariable biomarker model cautiously and complemented it with reduced sensitivity models.

**Table S5.** Pearson correlations among within-person biomarker components

| Within | ***APOB*** | ***APOE*** | ***APOJ*** | ***APOM*** | ***LPS*** |
| --- | --- | --- | --- | --- | --- |
| APOB | 1 |  |  |  |  |
| APOE | .527^**^ | 1 |  |  |  |
| APOJ | -.056 | -.056 | 1 |  |  |
| APOM | -.311^**^ | -.173 | -.454^**^ | 1 |  |
| LPS | .248^*^ | .183 | .217^*^ | -.270^*^ | 1 |

*Note****.*** *Within-person biomarker components represent deviations from each participant’s own mean across repeated assessments. Therefore, correlations reflect the degree to which intraindividual changes in one biomarker covary with intraindividual changes in another biomarker over time. APOB = apolipoprotein B; APOE = apolipoprotein E; APOJ = apolipoprotein J; APOM = apolipoprotein M; LPS = lipopolysaccharide. *= p < .05; **= p < .01.*

**Table S6.** Pearson correlations among between-person biomarker components

| *Between* | ***APOB*** | ***APOE*** | ***APOJ*** | ***APOM*** | ***LPS*** |
| --- | --- | --- | --- | --- | --- |
| *APOB* | *1* |  |  |  |  |
| *APOE* | *.086* | *1* |  |  |  |
| *APOJ* | *.348^**^* | *.303^**^* | *1* |  |  |
| *APOM* | *-.377^**^* | *-.103* | *-.661^**^* | *1* |  |
| *LPS* | *.372^**^* | *.150* | *.211^*^* | *-.184* | *1* |

*Note****.*** Between-person biomarker components represent each participant’s average biomarker level across repeated assessments. Therefore, correlations reflect associations between stable interindividual differences in biomarker levels. APOB = apolipoprotein B; APOE = apolipoprotein E; APOJ = apolipoprotein J; APOM = apolipoprotein M; LPS = lipopolysaccharide. *= p < .05; **= p < .01.

**2.3 Sensitivity analyses**

We conducted a series of reduced mixed-effects models as sensitivity analyses. All models included sex, age, education, time of abstinence, LPS (within), and LPS (mean). Each apolipoprotein and its person-level mean were then added separately.

**Table S7.** Reduced mixed-effects models including LPS and one apolipoprotein at a time

| **Model** | **Time of abstinence (SE), p** | **LPS (within) (SE), p** | **LPS (mean) (SE), p** | **Apo (within) (SE), p** | **Apo (mean) (SE), p** |
| --- | --- | --- | --- | --- | --- |
| Covariates + LPS | 1.365 (0.332),  p < .001 | -5.559 (2.272)  p = .018 | 2.547 (6.650)  p = .656 | - | - |
| Covariates + LPS + APOB | 1.635 (0.353),  p < .001 | -5.889 (2.264)  p = .012 | 0.910 (5.356)  p = .867 | 0.003 (0.004)  p = .489 | 0.004 (0.006)  p = .519 |
| Covariates + LPS + APOJ | 1.292 (0.393),  p = .002 | -5.955 (2.504)  p = .021 | 0.389 (5.576)  p = .945 | 0.010 (0.017)  p = .550 | 0.061 (0.039)  p = .131 |
| Covariates + LPS + APOM | 1.030 (0.405),  p = .014 | -5.804 (2.287)  p = .014 | 2.154 (5.783)  p = .713 | 0.125 (0.096)  p = .199 | -0.035 (0.105)  p = .742 |
| Covariates + LPS + APOE | 1.632 (0.372),  p < .001 | -5.420 (2.353)  p = .025 | 2.843 (5.697)  p = .622 | 0.053 (0.060)  p = .382 | -0.039 (0.156)  p = .800 |

*Note****.*** Reduced mixed-effects models including LPS and one apolipoprotein at a time showed that within-person LPS remained negatively associated with GCF across all model specifications, with estimates ranging from −5.420 to −5.955 and p-values ranging from .012 to .025.

- 1. **Baseline-to-follow-up comparisons**

We examine baseline-to-follow-up comparisons separately, we fitted additional reduced models comparing t=0 with t=1 and t=0 with t=2.

**Table S8.** Interval-specific reduced mixed-effects models comparing t=0–t=1 and t=0–t=2

|  | **t=0–t=1 Estimate (SE)** | ***p*** | **95% CI** |
| --- | --- | --- | --- |
| Intercept | 12.191 (4.769) | .017 | [2.387, 21.994] |
| Sex | 0.286 (1.269) | .824 | [-2.321, 2.892] |
| Age | -0.063 (0.077) | .418 | [-0.221, 0.095] |
| Education | 1.038 (1.135) | .368 | [-1.291, 3.368] |
| Time | 0.604 (0.655) | .364 | [-0.736, 1.945] |
| LPS (within) | -8.288 (2.912) | .008 | [-14.252, -2.325] |
| LPS (mean) | 2.652 (5.591) | .639 | [-8.834, 14.138] |
|  | **t=0–t=2 Estimate (SE)** | ***p*** | **95% CI** |
| Intercept | 7.954 (5.319) | .145 | [-2.918, 18.826] |
| Sex | 0.766 (1.393) | .586 | [-2.085, 3.618] |
| Age | -0.012 (0.087) | .894 | [-0.188, 0.165] |
| Education | 1.806 (1.248) | .159 | [-0.746, 4.358] |
| Time | 1.158 (0.408) | .008 | [0.323, 1.993] |
| LPS (within) | -7.657 (3.167) | .023 | [-14.152, -1.161] |
| LPS (mean) | 2.895 (6.082) | .638 | [-9.576, 15.366] |

*Note***.** Models were fitted separately for the t=0–t=1 and t=0–t=2 comparisons. Both models included sex, age, education, time, LPS within-person deviation, LPS person-level mean, and a random intercept for participant PS within-person deviation represents the deviation from each participant’s own mean LPS level, whereas LPS person-level mean represents the participant’s average LPS level across repeated assessments. LPS (within) represents the within-person deviation from each participant’s mean LPS level, whereas LPS (mean) represents the participant-level average LPS level. GCF = global cognitive functioning; LPS = lipopolysaccharide.

**3. SUPPORTING REFERENCES**

1. Asociación Americana de Psiquiatría (APA) (2014). *Manual Diagnóstico Y Estadístico De Los Trastornos Mentales DSM-5*. 5a. ed. Madrid: Editorial Médica Panamericana.
2. Babor, T. F., Higgins-Biddle, J. C., Saunders, J. B., & Monteiro, M. G. (2001). *The Alcohol Use Disorders Identification Test (AUDIT).* Guidelines for use in primary care.
3. Ferrando L, Bobes J, Gibert M, Soto M, Soto O. (1998). M.I.N.I. Mini International Neuropsychiatric Interview. Versión en español 5.0.0. DSM-IV. Instituto IAP, Madrid.
4. Beck AT, Steer RA, Brown GK. (1996). *Manual for the Beck Depression Inventory-II*. San Antonio, TX: Psychological Corporation.
5. Sanz, J., Navarro, M.E. y Vázquez, C. (2003). *Adaptación española del Inventario para la Depresión de Beck—II (BDI-II):* 1. Propiedades psicométricas en estudiantes universitarios. Análisis y notificación de conducta, 29: 239- 288.
6. Beck, AT & Steer, RA (2011). *Inventario de Ansiedad de Beck* (Adaptación española de Sanz, J.). Madrid: Pearson Educación.
7. Jurado-Barba R, Martínez A, Sion A, Álvarez-Alonso MJ, Robles A, Quinto-Guillen R, Rubio G. Development of a screening test for cognitive impairment in alcoholic population: TEDCA. Actas Esp Psiquiatr. 2017 Sep;45(5):201-17.
8. Rey A. Test de Copia y Reproducción de Memoria de Figuras Geométricas Complejas. Madrid: TEA Ediciones; 1997.
9. Bender L. Test Guestáltico Visomotor. Buenos Aires: Paidós; 2003.
10. Wechsler D, Pereña J. WMS-III: Escala de memoria de Wechsler III. Madrid: TEA Ediciones; 2004
11. Reitan R, Wolfson D. (1985). The Halstead–Reitan Neuropsychological Test Battery: Therapy and clinical interpretation. Tucson, AZ: Neuropsychological Press.
12. Wechsler D. Wechsler adult intelligence scale–Fourth Edition (WAIS–IV). San Antonio, TX: Pearson;2008.
13. Bates D, Maechler M, Bolker B, Walker S, Christensen R. H. B, Singmann H., ... 226 & Green, P. Package ‘lme4’. 2009. *URL http://lme4. r-forge. r-project. org*.
14. Halekoh, U., Højsgaard, S., Højsgaard, M. S., & Matrix, I. (2017). Package ‘pbkrtest’.
15. McNeish D. Small Sample Methods for Multilevel Modeling: A Colloquial Elucidation of REML and the Kenward-Roger Correction. Multivariate Behav Res. 2017 Sep-Oct;52(5):661-670.
